# Vertical scar reduction mammoplasty in massive macromastia: A prospective case series

**DOI:** 10.1016/j.jpra.2026.01.012

**Published:** 2026-01-16

**Authors:** Yahia Ahmed Alsiaghi, Mohammed Hasan Al Shoaibi, Mohaned Yahia Al-ajaly, Jehad Yahya Al Mortada, Aymen Mohammed Ghanem, Ahmed A․ S․ AL-Magedi

**Affiliations:** a Department of Surgery, Yemeni Council for Medical Specializations, Sana’a, Yemen; b Department of Plastic Surgery, Typical Police Hospital, Sana’a, Yemen; c Department of Plastic Surgery, Elite Hospital, Sana’a, Yemen; d Department of Surgery, Military General Hospital, Sana’a, Yemen; e Department of Surgery, Faculty of Medicine and Health Sciences, Sana’a University, Sana’a, Yemen; f Department of Surgery, 21 September University of Medical and Applied Sciences, Sana'a, Yemen

**Keywords:** Macromastia, Breast reduction, Vertical scar reduction mammoplasty, Superomedial pedicle, Complications, Patient satisfaction

## Abstract

**Background:**

Vertical scar superomedial pedicle breast reduction offers a shorter scar and improved breast projection, yet its safety and outcomes in cases of severe macromastia remain under discussion.

**Methods:**

A prospective case series was conducted from October 2022 to January 2025, including fifteen female patients (30 breasts) with suprasternal notch-to-nipple distance ≥30 cm or resection weight ≥750 g per breast. All underwent vertical scar reduction mammaplasty using a superomedial pedicle technique and were followed for 12 months.

**Results:**

The mean patient age was 33.7 ± 8 years, and the mean BMI was 28.3 ± 3.9 kg/m². Average resected tissue was 812.4 g (right) and 794.5 g (left). Common presenting symptoms were neck/back/shoulder pain (100%), shoulder grooving (93.3%), and social embarrassment (93.3%). Early complications were minimal, including wound dehiscence in two cases (13.3%), partial nipple-areolar necrosis in two cases (13.3%), and infection in one case (6.7%). No hematomas or seromas were observed. Late outcomes showed favorable results in breast shape (93.3%), volume (80%), scars (80%), and areola appearance (86.7%), with fair symmetry in 53.3% of cases. Patient satisfaction was high, with 80% rating the results as “very good” or “excellent.” Only 20% of patients required secondary revision. No statistically significant association was found between complications and smoking, obesity, or resection weight.

**Conclusion:**

Vertical scar superomedial pedicle breast reduction is a safe and effective technique for patients with severe macromastia. It provides excellent aesthetic outcomes, minimal complications, and high patient satisfaction, even in high-BMI populations. These findings support its role as a reliable first-line surgical option in the management of extensive breast hypertrophy.

## Introduction

The breast has traditionally been regarded as a symbol of femininity, and its morphology plays a central role in both body image and psychological well-being. Correction of breast deformities is therefore not only of physiological relevance but also of considerable psychological importance, with a proven positive impact on quality of life [1]. Beyond its brief nutritional and immunological role, the breast holds enduring significance in terms of sexuality and cultural ideals of attractiveness, although perceptions of size and shape vary across societies [2].

From an aesthetic standpoint, the ideal breast is proportional to body size, exhibits a mild degree of ptosis, assumes a teardrop-to-conical shape, and positions the nipple at the most anterior point [3,4]. Deviations from these parameters in terms of size, shape, or symmetry are not merely cosmetic concerns; they profoundly affect self-perception, emotional balance, and overall well-being [5]. Breast hypertrophy, in particular, constitutes a debilitating condition associated with both physical and psychosocial morbidity, frequently prompting patients to seek surgical intervention [6].

Reduction mammaplasty aims to decrease breast volume, restore proportionality, improve contour, and alleviate symptoms associated with hypertrophy. Evidence also suggests a potential reduction in breast cancer risk following reduction procedures [7]. Common indications include musculoskeletal pain, bra strap grooving, limitations in daily activity, intertriginous dermatitis, and, in some cases, respiratory compromise.

Several surgical approaches have been described, with the inverted T and vertical scar techniques being the most widely utilized [8]. A significant disadvantage of the inverted T pattern is the high scar burden, which may be a consideration for patients who tend to develop keloid or hypertrophic scarring. It also runs the risk of giving a boxy breast appearance if the medial and lateral incisions do not curve up against the breast enough [9]. The vertical scar technique, conversely, offers advantages in terms of reduced scarring, improved breast projection, and durable long-term aesthetic outcomes, with high patient acceptance rates [10].

Historically, vertical scar reduction was considered unsuitable for severe macromastia or marked ptosis due to concerns over pedicle length, nipple transposition, and the risk of compromising nipple–areola complex (NAC) vascularity [11,12]. Traditional guidelines discouraged its use when resection volumes exceeded 1000 g or when nipple transposition exceeded 10 cm. However, more contemporary evidence indicates that the Lejour vertical technique achieves satisfactory outcomes even in cases of gigantomastia, with complication rates comparable to those of inverted T approaches [13]. While minor revisions for skin redundancy are more frequent, these are generally regarded as staged refinements rather than true complications. The adoption of vertical scar techniques continues to increase, despite perceptions of greater technical difficulty [14].

Recent comparative studies have demonstrated that both inverted T and vertical scar reductions are safe and effective. Notably, vertical scar approaches have been associated with lower rates of wound dehiscence and infection, fewer scar-related complications, and shorter operative times [15].

Accordingly, this study aims to evaluate the safety, complication rates, and patient satisfaction associated with vertical scar superomedial pedicle breast reduction in women with severe macromastia or significant ptosis.

## Material and methods

This prospective case series was conducted in the Department of Plastic and Reconstructive Surgery at Elite and European Hospitals, Sana’a City, Yemen, and included all patients meeting the inclusion criteria between October 20, 2022, and January 1, 2025. Fifteen female patients with severe macromastia or significant breast ptosis underwent vertical scar superomedial pedicle reduction mammoplasty.

The procedures were performed by three senior board-certified plastic surgeons, assisted by two plastic-surgery residents. All surgeons adhered to the same standardized superomedial vertical technique to ensure uniformity.

The study protocol was approved by the Ethics Committee of the Yemeni council for Medical Specializations (Approval No. IRB/YBMS/2022/047). All procedures adhered to the ethical principles of the Declaration of Helsinki (1964) and relevant national and institutional guidelines. Written informed consent, including permission for academic use of patient data, was obtained from all participants after a thorough explanation of the procedure, risks, benefits, and expected outcomes. No animals were involved in this study. The manuscript was checked against the Strengthening the Reporting of Observational Studies in Epidemiology (STROBE) checklist [16] (Supplemental Appendix).

### Inclusion and exclusion criteria

Inclusion criteria: Female patients with a suprasternal notch (SN)-to-nipple-areolar complex (NAC) distance ≥ 30 cm, anticipated breast tissue resection ≥ 750 g per breast, or a diagnosis of massive macromastia or severe breast ptosis requiring surgical intervention.

Exclusion criteria: SN-to-NAC distance ≤ 29 cm, anticipated resection < 750 g per breast, significant comorbidities (e.g., diabetes mellitus, collagen disorders, chronic corticosteroid use), medical unfitness for surgery, active intertrigo or other breast skin abnormalities, and patients requiring unilateral breast reduction.

### Instruments and materials

The following instruments and materials were used for patient evaluation and surgical planning:

Digital weighing scale: For measuring the weight of resected breast tissue.

Measuring tape: For recording the Suprasternal Notch (SN) to nipple-areola complex distance and other breast dimensions.

Surgical skin marker: For preoperative skin markings.

Standard surgical instruments: For vertical scar breast reduction procedures.

### Preoperative assessment

a) History taking: A comprehensive history was obtained, including demographic data (age, marital status) and medical comorbidities (diabetes mellitus, collagen disorders, corticosteroid use, etc.). The main presenting complaints were assessed in detail, emphasizing physical symptoms (neck, back, and shoulder pain; intertriginous rash; shoulder grooving; asymmetry; upper limb paresthesia), sleep disturbance, and social or psychological distress. The vertical scar pattern and expected scar length were explained and demonstrated to all patients.
b) Clinical examination: Anthropometric measurements (weight, height, BMI) were recorded. Each patient underwent a detailed breast examination in upright, sitting, leaning-forward, and supine positions to evaluate asymmetry, ptosis, and the presence of any breast masses or abnormalities. Standardized preoperative photographs were obtained and reviewed with the patients.
c) Investigations: Routine preoperative investigations included a complete blood count, coagulation profile, liver and kidney function tests, fasting blood glucose, chest radiograph, and electrocardiogram.

### Preoperative and intraoperative marking

**Figure 1:**
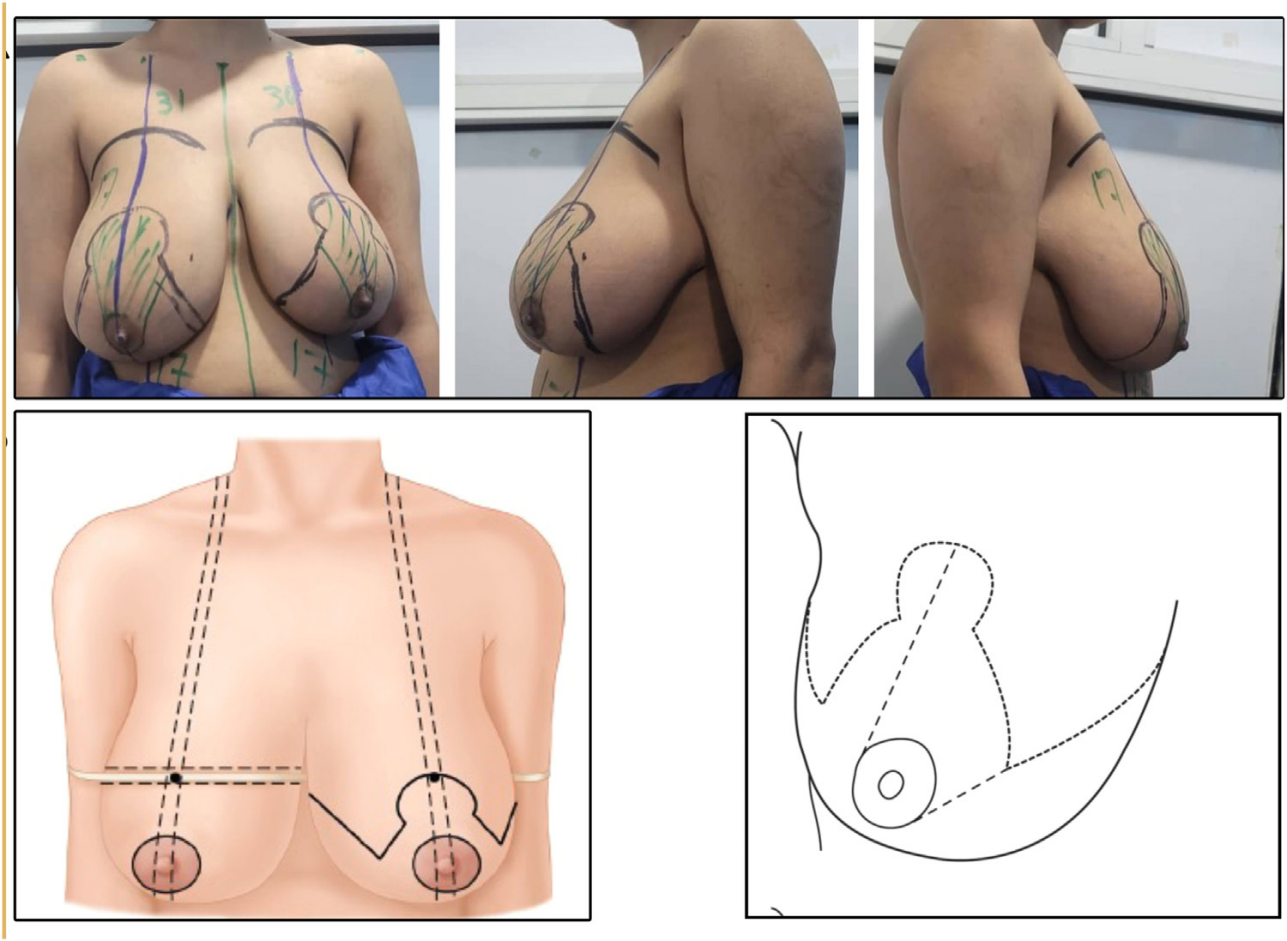
Preoperative and intraoperative marking. Marking of vertical scar reduction mammoplasty; Identification of new nipple position [22]; Marking and design of superomedial pedicle.

The following marking had been taken (Figure 1):

1. Midline from the SN to the umbilicus.
2. Breast meridian (from the midclavicular point to the midbreast, bisecting the breast into two halves).
3. Position of the inframammary fold (IMF) (2 cm from the midline and anterior axillary line).
4. Nipple-areolar complex diameter.
5. Internipple distance.
6. IMF to the inferior border of the NAC distance length.
7. Grade of ptosis.
8. New nipple position is marked (The Pitanguy point, or the anterior projection of the IMF onto the breast meridian, is marked by direct palpation or using a flexible ruler positioned under the breast [17,18]).
9. Distance of the two vertical limbs (6–7 cm).
10. Connecting the lowest points of vertical limbs to the IMF medially and laterally.
11. A 4.5 cm diameter circle is drawn within the areola using the cookie cutter.
12. Pedicle design was done (The superomedial pedicle is designed extending from the keyhole pattern to the bottom end of the medial vertical limb. The base of the pedicle extends partially in the keyhole and entirely in the vertical limb, leaving a 1–2 cm border around the areola. The width of the pedicle varies between 8 and 10 cm.
13. New IMF mark again when the patient is in the supine position.
14. Comparing the measurements on both sides.
15. Resection weight per breast.

### Surgical technique

All patients underwent vertical scar reduction mammoplasty using the superomedial pedicle technique under general anesthesia. Patients were positioned supine with both shoulders abducted to 90°.

### Superomedial pedicle selection and de-epithelialization

A breast tourniquet was applied to maintain skin tension and minimize bleeding. The NAC was marked with a 45 mm diameter circle centered on the nipple. A superomedial dermoglandular pedicle was outlined, extending from the midpoint of the mosque dome roof to the medial blocking triangle, leaving a 2.5 cm border around the NAC.

**Figure 2:**
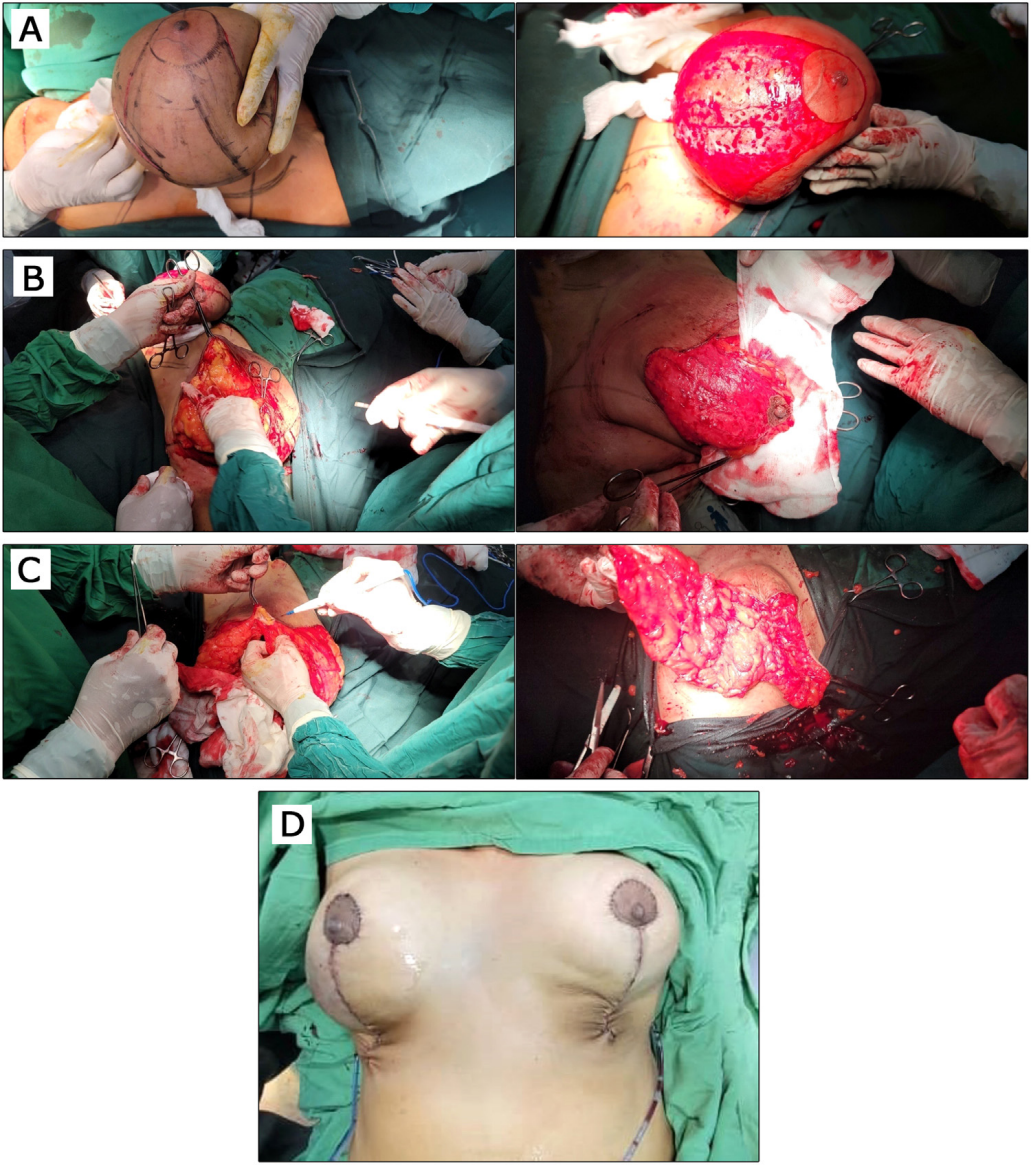
Surgical Technique. A: Mosque dome skin marking pattern with superomedial pedicle; Superomedial pedicle de-epithelialized. B: Medial breast tissue is left intact, preserving medial fullness; Partial thickness superomedial pedicle is 2.5 cm thick. C: Excision of breast tissue between the end of the vertical wound and the inframammary crease to prevent a dog-ear from forming; Parenchymal pillar sutures. D: The nipple-areolar complex has been inset, a vacuum drain was put, and the vertical wound was closed by subcuticular suture.

With the tourniquet in place, de-epithelialization of the dermoglandular pedicle was facilitated by increased skin tension. The NAC and pedicle were marked, and the deep dermis was preserved to protect superficial vascular structures (Figure 2A).

### Excision of breast tissue

Skin, fat, and glandular tissue were excised en bloc along preoperative markings. The dermoglandular pedicle was raised to a depth of 2.5 cm. Medial and lateral parenchymal pillars were developed through vertical limb incisions extending to just above the pectoralis fascia, maintaining a uniform 2.5 cm thickness. The medial pillar preserved medial fullness and improved final breast contour. The inferior junction of the vertical limbs formed a “V” shape, joining the medial and lateral dissections, which were then carried to the chest wall. Dissection proceeded inferior-to-superior along the pectoralis fascia, leaving a thin layer of tissue to reduce bleeding and postoperative pain. The superior flap was completed beneath the dermoglandular pedicle to maintain even flap thickness (Figure 2B). Excess inferior tissue near the inframammary fold was thinned to prevent dog-ear formation (Figure 2C).

### Liposuction

Liposuction of the lateral thoracic compartment was performed to contour the lateral breast and axillary regions. Subaxillary rolls were addressed selectively. In most cases, lateral liposuction through the same vertical access improved the contour. When patients specifically requested formal excision of axillary rolls, a Wise-pattern approach was preferred.

### Breast shaping

Breast projection and contour were achieved by reapproximating the medial and lateral parenchymal pillars with two or more inverted #1 Vicryl sutures placed through the breast capsule. These sutures prevent pseudoptosis and maintain long-term projection (Figure 2C).

### Wound closure

The NAC was advanced or rotated into position and inset before vertical closure. If tension occurred, small superficial dermal incisions superior to the blocking triangles facilitated in setting. The vertical incision was closed in layers (Figure 2D). The slight obliquity of the vertical closure is intentional, designed to avoid inferior dog-ear formation and to accommodate lateral breast fullness. The closure consistently terminates above the IMF without crossing it.

### Postoperative management

A. Immediate postoperative care

• Early mobilization is encouraged to prevent deep vein thrombosis.
• Adequate analgesic protocols for pain management.
• Hospital stay duration documentation.
• Prophylactic antibiotic administration for 7 days postoperatively.

B. Wound care protocol

• Light wound dressing with soft dressing at the nipple-areolar complex.
• Immediate postoperative assessment of nipple and areolar viability.
• Daily wound inspection and dressing changes as indicated.
• Suction drain management with removal after 3–5 days.
• Suture removal: peri‑areolar sutures at 10 days, vertical sutures at 14 days.

C. Follow-up

Systematic follow-up was conducted at predetermined intervals:

• Early follow-up: 1, 2, and 4 weeks postoperatively.
• Long-term follow-up: 3, 6, and 12 months postoperatively.

D. Outcome measures

• Patient satisfaction assessment through direct interview.
• Postoperative complications documentation.
• Aesthetic outcome evaluation using the Ferreira classification scheme.
• Revision surgery requirements recording.

### Data collection

Data were prospectively collected for all patients with massive macromastia or severe ptosis who underwent vertical scar superomedial pedicle reduction mammoplasty (VSMP). Recorded variables included age, weight, height, BMI, marital status, and risk factors such as smoking, diabetes, steroid use, and obesity. Presenting symptoms (neck, back, or shoulder pain; intertrigo; shoulder grooving; asymmetry; paresthesia; sleep disturbance; or social embarrassment) were documented. Pre- and postoperative photographs were obtained. Surgical data included preoperative measurements (SN—N distance, nipple–IMF distance, areolar diameter, internipple distance), intraoperative data (resected tissue weight per breast), and postoperative data (drain removal time and hospital stay). Early complications (hematoma, seroma, dehiscence, infection, edge necrosis, NAC or fat necrosis) and late outcomes were assessed using the extended Ferreira classification (volume, shape, symmetry, areola, and scar). Revision procedures and patient satisfaction were recorded. Follow-up was performed daily for 7 days, weekly for 1 month, and at 3, 6, and 12 months. All data were collected by the operating surgeon and stored securely to maintain confidentiality.

### Statistical analysis

Data analysis was performed using SPSS (Statistical Package for the Social Sciences), version 23, for Microsoft Windows. Descriptive statistics were calculated, including means, standard deviations, medians, and ranges for continuous variables, as well as frequencies and percentages for categorical variables when appropriate.

## Results

**Figure 3:**
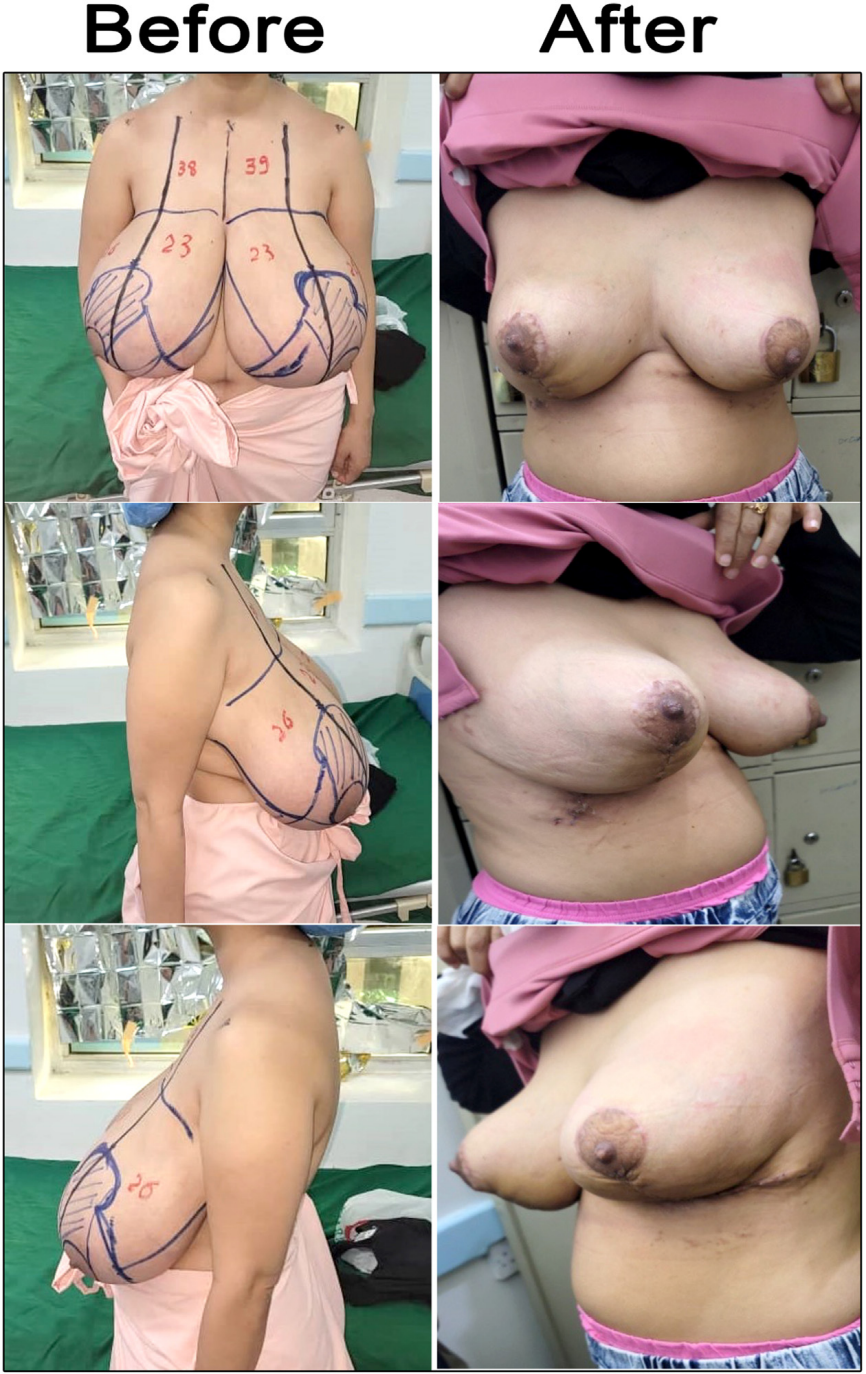
Case 1 before and after 6 months of vertical scar superomedial reduction mammoplasty. A 35-year-old single patient presented with macromastia. BMI was 31 kg /m², the SSN—NAC distances were 38 cm right side and 39 cm left side, and she underwent vertical scar superomedial pedicle reduction mammoplasty. The weight of resected breast tissue was 1660 g from the right side and 1802 g from the left side.

**Figure 4:**
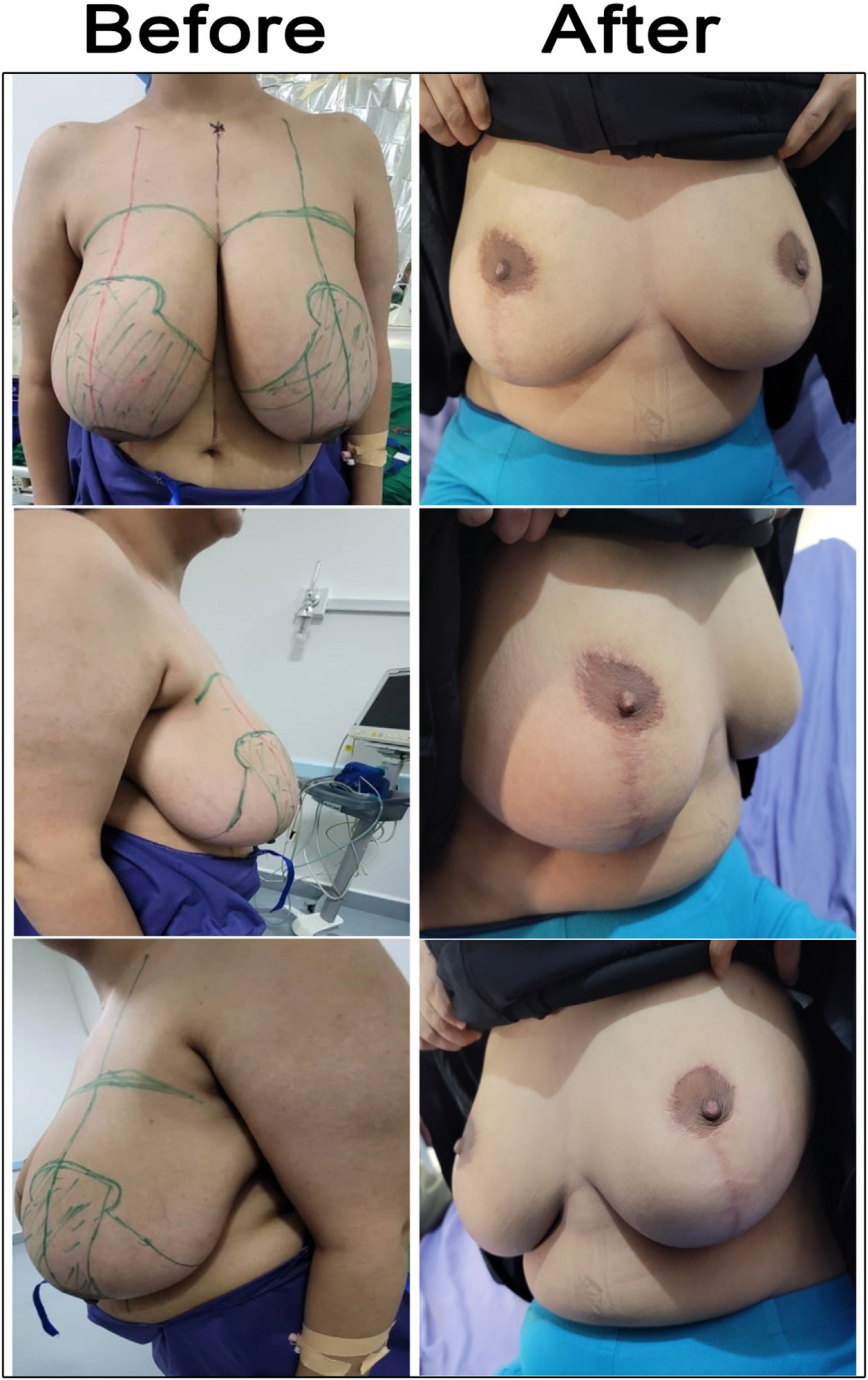
Case 2 before and after 6 months of vertical scar superomedial reduction mammoplasty. A 27-year-old married patient came complaining of macromastia. The BMI was 32 kg/m², and SSN—NAC distances were 37 cm on both the right and left sides. She underwent a vertical scar superomedial pedicle reduction mammoplasty. The weight of resected breast tissue was 750 g from the right side and 900 g from the left side.

**Figure 5:**
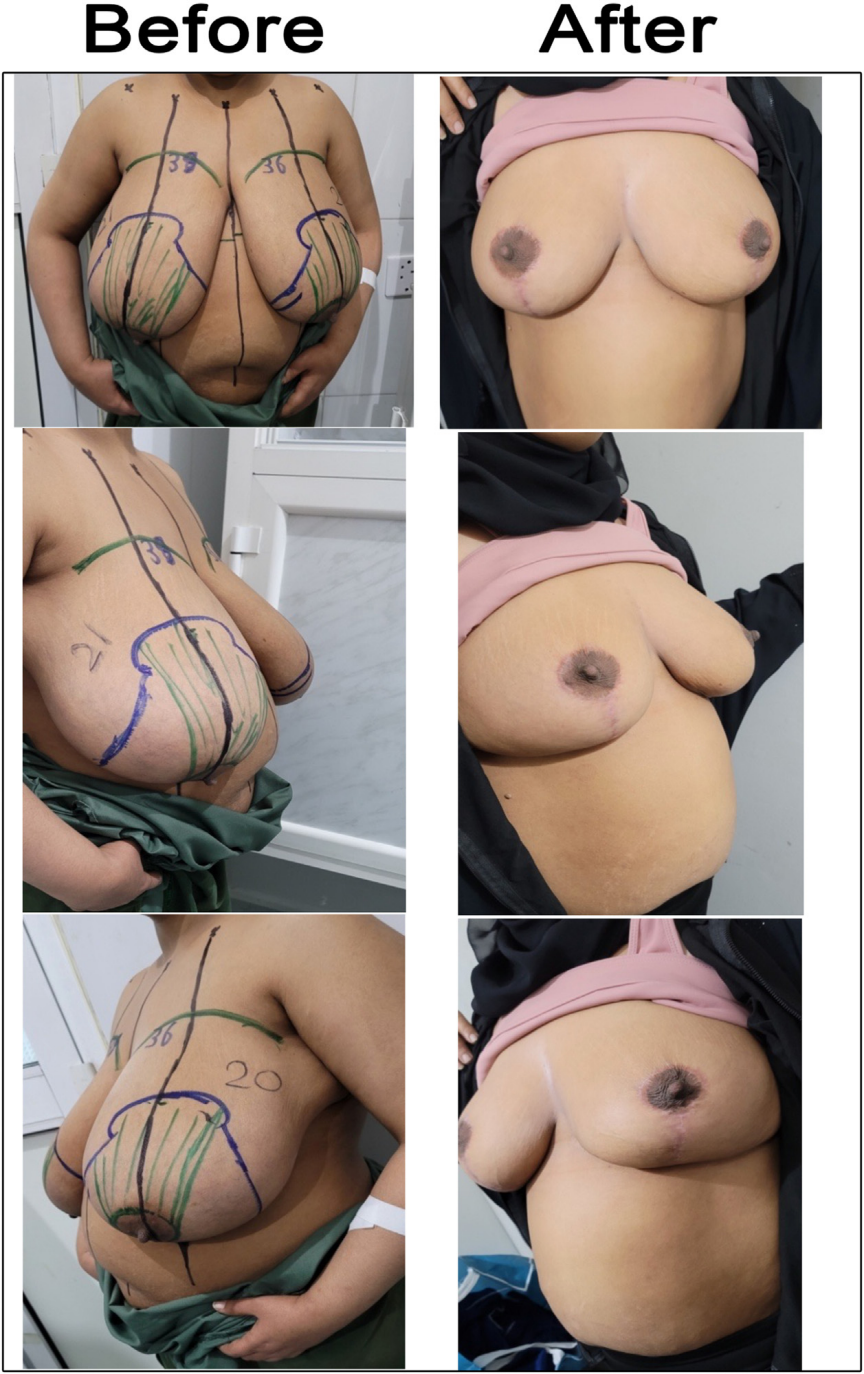
Case 3 before and after 6 months of vertical scar superomedial reduction mammoplasty. A 36-year-old married patient presented with macromastia, and the BMI was 31 kg/m². SSN—NAC distances were 39 cm on the right side and 36 cm on the left side. She underwent vertical scar superomedial pedicle reduction mammoplasty. The weight of resected breast tissue was 1241 g from the right breast and 989 g from the left breast.

**Figure 6:**
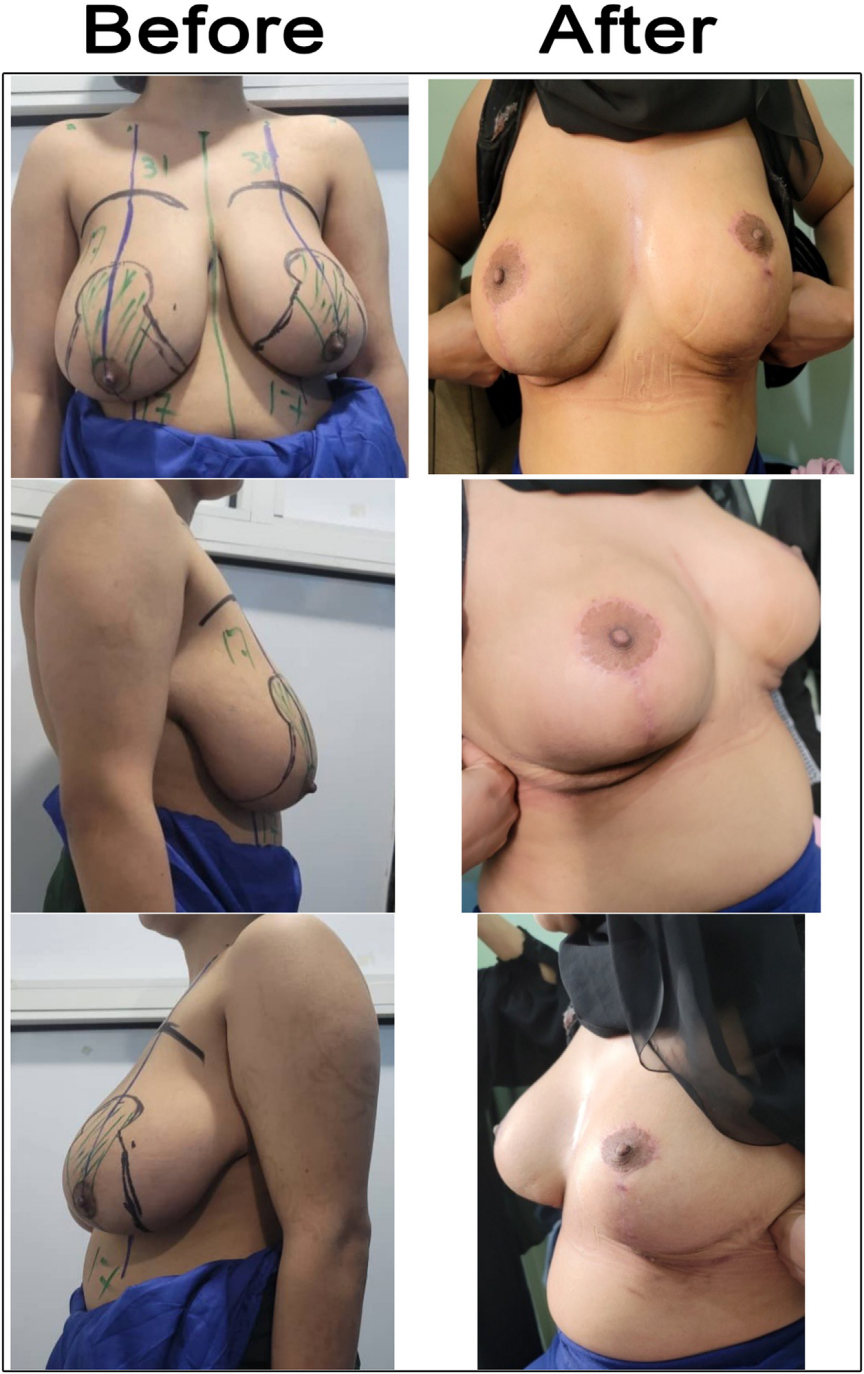
Case 4 before and after 6 months of vertical scar superomedial reduction mammoplasty. A 25-year-old married patient presented with macromastia, and the BMI was 25.3 kg/m². SSN—NAC distances were 31 cm on the right side and 30 cm on the left side. She underwent vertical scar superomedial pedicle reduction mammoplasty. The weight of resected breast tissue was 750 g from the right breast and 500 g from the left breast.

**Figure 7:**
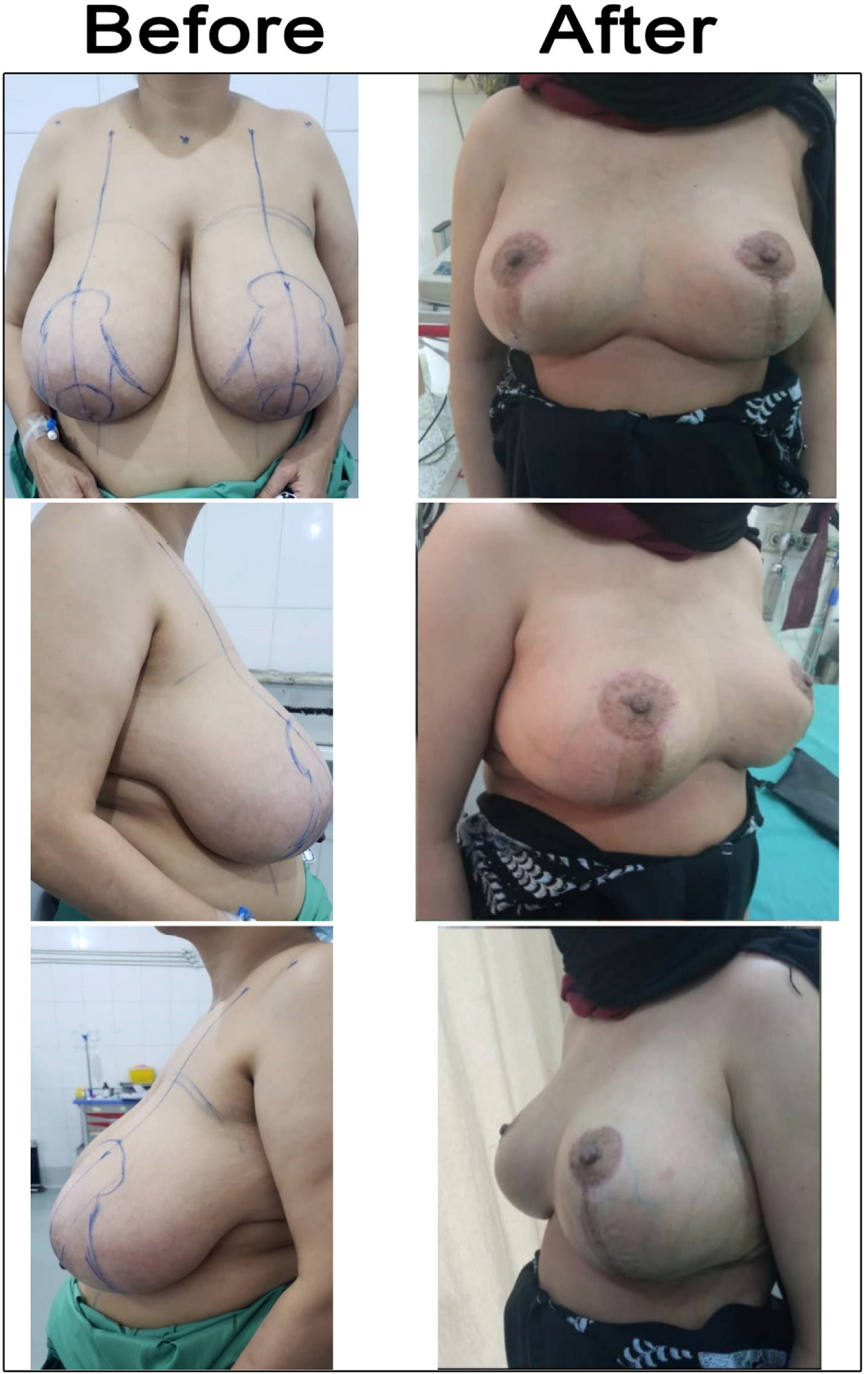
Case 5 before and after 6 months of vertical scar superomedial reduction mammoplasty. A 39-year-old married patient presented with macromastia, BMI 29.6 kg/m². The SSN—NAC distances were 36 cm on both the right and left sides. She underwent vertical scar superomedial pedicle reduction mammoplasty. The weight of resected breast tissue was 1100 g from the right breast and 1050 g from the left side.

This prospective case series study, conducted from October 20, 2022, to January 1, 2025, included all patients attending our clinic who complained of symptomatic massive macromastia or severe ptosis and sought help for reduction mammaplasty. We used the vertical scar superomedial pedicle reduction technique. Patients with unilateral reduction were excluded from our study. Fifteen patients were included, which is a total of 30 breasts (Figure 3, Figure 4, Figure 5, Figure 6, Figure 7). Cases before and after 6 months of vertical scar superomedial reduction mammoplasty.

### Demographic characteristics

The ages of the 15 participants in the study ranged from 24 to 50 years, with a mean age of 33.73 years ± 8.01 years. The majority of the patients (53.3%) were in the 30–39 age group. The analysis of the age distribution reveals that the largest cohort of patients seeking reduction mammaplasty was between 30 and 39 years old, representing over half of the study sample. The second most common age group was patients in their twenties (33.3%). Participants aged 40 and above constituted a smaller portion of the sample (13.4%). The study predominantly included married individuals (66.7%, *n* = 10), with minorities being single (20.0%, *n* = 3) or divorced (13.3%, *n* = 2). No widowed participants were recorded.

A minority of participants reported current smoking (13.3%, *n* = 2). Notably, these patients denied smoking preoperatively, but their relatives confirmed postoperative smoking behavior. No participants reported pre-existing chronic diseases or steroid use. The mean BMI was 28.34 ± 3.87 kg/m², with a range of 20.70 to 33.30 kg/m². According to BMI classification: Obesity (BMI ≥ 30): 46.7% (*n* = 7); Overweigh (25 ≤ BMI < 30): 33.3% (*n* = 5); Normal weight (BMI < 25): 20.0% (*n* = 3) (Table 1).

**Table 1:** Participants' demographic characteristics.

Demographic characteristics	Number of patients (%)
Age group (years)	
20–29	5 (33.3%)
30–39	8 (53.3%)
40–49	1 (6.7%)
50–59	1 (6.7%)

Marital status	
Single	3 (20.0%)
Married	10 (66.7%)
Divorced	2 (13.3%)
Widow	0

Risk factors and comorbidities	
Smoking	2 (13.3%)
Chronic diseases	0
Steroid use	0
Obesity (BMI ≥ 30)	7 (46.7)
Overweight (25 ≤ BMI < 30)	4 (33.3)

### Clinical presentation and symptoms

All participants (100%, *n* = 15) reported experiencing pain in the neck, back, and shoulders. The majority experienced shoulder grooving and social embarrassment (93.3%, *n* = 14 each). Breast asymmetry was reported in 60.0% (*n* = 9) of cases, while upper limb paresthesia affected 53.3% (*n* = 8). Sleep compromise was noted in 46.7% (*n* = 7) of participants, and intertriginous rash in 40.0% (*n* = 6) of participants (Table 2).

**Table 2:** Presenting symptoms among study participants.

Symptoms	Present	Absent
	N (%)	N (%)
Neck pain	15 (100.0)	0
Back pain	15 (100.0)	0
Shoulder pain	15 (100.0)	0
Shoulder grooving	14 (93.3)	1 (6.7)
Social embarrassment	14 (93.3)	1 (6.7)
Breast asymmetry	9 (60.0)	6 (40.0)
Upper limb paresthesia	8 (53.3)	7 (46.7)
Sleep compromise	7 (46.7)	8 (53.3)
Intertriginous rash	6 (40.0)	9 (60.0)

### Anthropometric and surgical parameters

Mean participant weight was 67.00 ± 7.83 kg (range:54–78 kg),with a mean height of 154.07 ± 4.20 cm (range: 147–162 cm). The mean sternal notch to nipple distance was 33.57 cm (right: 33.73 ± 3.01 cm; left: 33.40 ± 2.95 cm). Mean nipple to IMF distance was 18.57 cm (right: 18.70 ± 2.46 cm; left: 18.43 ± 2.50 cm). Mean areolar diameter was 7.90 cm (right: 8.00 ± 1.79 cm; left: 7.80 ± 1.88 cm), with a mean internipple distance of 30.20 ± 3.65 cm.

Regarding surgical parameters, high variability was observed in the weights of resected breast tissue (right: 812.40 ± 390.20 g, range 222–1660 g; left: 794.53 ± 410.64 g, range 208–1802 g). Mean drain removal time was 4.47 ± 0.83 days (range: 3–5 days), and mean hospital stay was 1.13 ± 0.35 days (range: 1–2 days) (Table 3).

**Table 3:** Descriptive statistics of anthropometric and surgical parameters.

Parameter	Mean ± SD	Range	Min	Max
Age (years)	33.73 ± 8.01	26.00	24.00	50.00
Weight (kg)	67.00 ± 7.83	24.00	54.00	78.00
Height (cm)	154.07 ± 4.20	15.00	147.00	162.00
BMI (kg/m²)	28.34 ± 3.87	12.60	20.70	33.30
SN—N distance - Right (cm)	33.73 ± 3.01	10.00	29.00	39.00
SN—N distance - Left (cm)	33.40 ± 2.95	10.00	29.00	39.00
Nipple to IMF distance - Right (cm)	18.70 ± 2.46	9.00	14.00	23.00
Nipple to IMF distance - Left (cm)	18.43 ± 2.50	9.00	14.00	23.00
Areolar diameter - Right (cm)	8.00 ± 1.79	7.00	5.00	12.00
Areolar diameter - Left (cm)	7.80 ± 1.88	8.00	5.00	13.00
Internipple distance (cm)	30.20 ± 3.65	15.00	22.00	37.00
Resected weight - Right (g)	812.40 ± 390.20	1438.00	222.00	1660.00
Resected weight - Left (g)	794.53 ± 410.64	1594.00	208.00	1802.00
Drain removal time (days)	4.47 ± 0.83	2.00	3.00	5.00
Hospital stays (days)	1.13 ± 0.35	1.00	1.00	2.00

BMI, body mass index; IMF, inframammary fold; SN—N distance, suprasternal notch to nipple distance.

### Surgical outcomes and complications

Overall, there was a low incidence of early complications (≤ 30 days postoperatively). Partial wound dehiscence occurred in two cases (13.3%), partial nipple-areolar complex necrosis in two cases (13.3%), and wound infection in one case (6.7%). Nipple sensation was preserved in all patients, with no reported cases of complete loss. Two patients reported mild transient hypoesthesia that resolved within 3 months. No cases of hematoma or seroma were observed.

Aesthetic outcomes (Ferreira's standardized scheme): Final aesthetic results were predominantly favorable across all parameters. A good volume was achieved in 80.0% (*n* = 12) of patients, with 20.0% (*n* = 3) having a fair volume. The shape was rated as good in 93.3% (*n* = 14) and fair in 6.7% (*n* = 1). Symmetry showed the most variability, with 46.7% (*n* = 7) achieving good symmetry and 53.3% (*n* = 8) fair symmetry. Areolar outcomes were good in 86.7% (*n* = 13) and fair in 13.3% (*n* = 2). Scar quality was good in 80.0% (*n* = 12) and fair in 20.0% (*n* = 3). No poor outcomes were recorded in any category (Table 4).

**Table 4:** Complications and aesthetic outcomes.

Variables	Number of patients (%)		
Complication	Present		
Hematoma	0		
Seroma	0		
Wound dehiscence	2 (13.3)		
Wound infection	1 (6.7)		
Partial areolar necrosis	2 (13.3)		

Aesthetic parameter	Poor n (%)	Fair n (%)	Good n (%)

Volume	0	3 (20.0)	12 (80.0)
Shape	0	1 (6.7)	14 (93.3)
Symmetry	0	8 (53.3)	7 (46.7)
Areola	0	2 (13.3)	13 (86.7)
Scars	0	3 (20.0)	12 (80.0)

### Patient-reported outcomes

Patient satisfaction was generally high, with 46.7% (*n* = 7) reporting excellent satisfaction and 33.3% (*n* = 5) reporting very good satisfaction. Only 6.7% (*n* = 1) reported good satisfaction, while 13.3% (*n* = 2) expressed poor satisfaction with the final results.

Surgical revision: The majority of patients (80.0%, *n* = 12) required no revision surgery. Secondary revisions were performed in 20.0% (*n* = 3) of cases, while no tertiary revisions were necessary (Table 5).

**Table 5:** Patient satisfaction and surgical revision.

Satisfaction level	Number of patients (%)
Poor	2 (13.3)
Good	1 (6.7)
Very good	5 (33.3)
Excellent	7 (46.7)

Surgical revision	

Secondary revision	3 (20.0)
Tertiary revision	0
No revision required	12 (80.0)

### Statistical analysis of risk factors

No statistically significant association was found between smoking or obesity and any specific early or late complications (*P*-values > 0.05) (Table 6). Similarly, the Mann-Whitney U test revealed no statistically significant difference in resected breast tissue volume between patients requiring secondary revision and those not requiring revision (*P* > 0.05 for both right and left breast volumes) (Table 7). Follow-up compliance was 100% at 1 month, 93.3% at 3 months, 80% at 6 months, and 73.3% at 12 months.

**Table 6:** Correlation between risk factors and complications.

Complication type	Correlation coefficient (rho)	*P*- value	Interpretation
Correlation between smoking and complications			
Early complications (total score)	0.260	0.349	Weak positive correlation, not significant
Late complications (total score)	−0.196	0.484	Weak negative correlation, not significant
Total complications	−0.071	0.801	Very weak negative correlation, not significant

Correlation between obesity and complications (Spearman's correlation)			

Early complications (total score)	0.221	0.428	Weak positive correlation, not significant
Late complications (total score)	−0.367	0.178	Moderate negative correlation, not significant
Total complications	−0.097	0.730	Very weak negative correlation, not significant

**Table 7:** Relationship between resected breast tissue volume and revision surgery.

Variable	Mean rank	U value	Z value	*P*-value
Right breast volume				
Revision required	11.33	8.000	−1.446	0.148
No revision	7.17			

Left breast volume				

Revision required	12.17	5.500	−1.806	0.071
No revision	6.96			

## Discussion

Breast reduction mammoplasty aims to relieve the physical and psychological burden of macromastia by reducing breast volume, reshaping the mound, and preserving nipple-areola complex (NAC) viability for an aesthetic outcome [3]. The traditional inverted-T (Wise pattern) technique remains reliable for large reductions but carries drawbacks such as extensive scarring, risk of hypertrophic or keloid scars, “boxy” breast shape, and complications like T-junction necrosis and bottoming-out deformity [8]. In contrast, the VSMP has gained popularity for its reduced scar burden, enhanced projection, and natural conical shape. The Lassus-type vertical scar technique employs a superomedial or medial pedicle to maintain robust NAC vascularity while excising tissue in a vertically oriented pattern [19]. Studies have demonstrated lower rates of wound dehiscence and infection, shorter operative time, and faster recovery compared to the traditional inverted-T method [20]. Although the Wise pattern reliably addresses redundant skin, our choice of the vertical superomedial approach is supported by several factors. Vertical techniques demonstrate reduced T- junction complications, improved long-term projection, better scar acceptance particularly in high-BMI populations, and lower wound-related morbidity. These advantages are especially relevant in resource-limited settings where wound care constraints favor techniques with fewer tension points.

The present prospective series further validates the efficacy and safety of the vertical scar superomedial pedicle technique in patients with severe macromastia and significant ptosis, a population often considered high risk due to large resection volumes, long pedicles, elevated BMI, and limited operative resources. Based on our experience and consistent with contemporary literature, the superomedial vertical approach was applied safely in patients with SN–N distances up to 39 cm, resection weights up to 1800 g, and BMI values up to 33. These practical thresholds informed patient selection and preoperative counseling. Our cohort of 15 patients (30 breasts) represents this challenging demographic, with a mean suprasternal notch–to–nipple (SN—N) distance of 33.57 cm and mean resection weights exceeding 750 g per breast.

The mean age of our patients was 33.7 years (range, 24–50), comparable to that reported by Serra et al. [21]. The mean BMI was 28.3 kg/m², with 46.7% classified as obese and 33.3% as overweight. These findings align with those of Copeland-Halperin et al. [14], who found that VSMP remains safe and effective in higher-BMI cohorts, although obesity may predispose individuals to delayed wound healing. Despite this, our series showed low complication rates, supporting the safety of VSMP even among overweight and obese patients. Although smoking and obesity are well-known risk factors for wound complications, neither parameter independently predicted adverse outcomes in our study, consistent with Hall-Findlay’s [22] assertion that careful surgical technique—including meticulous hemostasis, tension-free closure, and appropriate drain management—can mitigate traditional risk factors.

Our median SN—N distance (33.6 cm) places our patients at the upper limit of high-risk criteria identified by Mahrhofer et al. [23], who noted an increased risk of NAC morbidity when the distance exceeds 36.5 cm. Nevertheless, successful nipple transposition with full vascular preservation was achieved in all cases, supporting the findings of Lista and Ahmad [24] and Neaman et al. [25] that the superomedial pedicle provides reliable perfusion even in extensive reductions. Mean resection weights were 812.4 g (right) and 794.5 g (left), with a maximum resection of 1802 g—well within the range associated with increased complications according to Mahrhofer et al. [23]. Despite these large resections, only minor complications occurred: partial areolar necrosis (13.3%), superficial wound dehiscence (13.3%), and minor infection (6.7%). Partial NAC necrosis occurred in two cases (13.3%). Neither patient was a smoker. Contributing factors included large pedicle length (>34 cm SN–N), thick pedicle base, and increased dermal tension at inset. Both cases healed secondarily without functional loss. There were no cases of total NAC necrosis, hematoma, or systemic morbidity. These findings reaffirm the vascular stability of the superomedial pedicle, as also highlighted by Selamioğlu and Ağdoğan [26] and Sak et al. [13], who demonstrated that free nipple grafting is unnecessary when pedicle perfusion is well maintained, even in gigantomastia.

Aesthetic and functional outcomes were excellent. Using the Ferreira classification system, 93.3% of patients achieved good or excellent breast shape, 80% satisfactory scar quality, and 86.7% satisfactory areolar appearance. These results are comparable to those of La Padula et al. [27], who emphasized stable upper-pole projection and a conical contour, and Panopoulou et al. [28], who achieved 100% good or excellent breast form in patients with gigantomastia. A recognized concern in large reductions is the potential for gradual inferior pole descent or pseudoptosis due to stretched parenchymal support. Although our 12-month outcomes did not show significant bottoming-out, longer follow-up with standardized photography is required to fully evaluate long-term stability.

Although satisfaction after breast reduction is generally high in the literature (80% rating their results as “very good” or “excellent”), our inclusion of patient-reported data provides confirmation that the vertical superomedial technique remains well-accepted even in large-breasted patients, consistent with outcomes reported by Ahmad and Lista [29] and Lonie et al. [30].

The 20% revision rate in our series reflects minor elective refinements rather than complications as also suggested by Sak et al. [13]. Comparative studies report that vertical techniques may have slightly higher secondary refinement rates than Wise pattern reductions, primarily related to skin redundancy. However, these revisions were elective, performed under local anesthesia, and provided at no additional cost to patients. They were typically offered after 6–9 months, once breast shape had stabilized. Although the revision rate is higher compared with the Wise pattern, these were minor touch-ups rather than complication-related procedures, and overall patient satisfaction remained high.

Functional improvement was substantial. Nearly all patients reported relief from shoulder, back, and neck pain, with 93.3% experiencing improved shoulder mobility and enhanced social confidence. Additional improvements included the resolution of intertrigo, sleep disturbances, and cervicodorsal discomfort, paralleling findings from Gonzalez et al. [7], Spector et al. [31], and Lonie et al. [30], who reported similar symptom relief following reduction mammaplasty.

From a health service perspective, VSMP demonstrated practical and economic advantages, with a mean operative time of 97 min, mean drain removal time of 4.47 days, and mean hospital stay of 1.13 days. These findings align with Tanas and Tanas’s [32] meta-analysis, which reported shorter operative times for superomedial compared to inferior pedicle techniques, and with Li et al. [33], who emphasized efficiency in operating room utilization through anthropometric-based planning. Such benefits are particularly valuable in resource-limited environments, underscoring the pragmatic appeal of the VSMP technique.

Nevertheless, this study has several limitations. The small sample size and single-center design limit the generalizability of our findings. Postoperative follow-up compliance was variable, which hindered standardized long-term photographic assessment. The absence of a control group precluded direct comparison with alternative pedicle techniques, and the relatively short follow-up period limited evaluation of scar maturation and long-term breast shape. Finally, smoking status was self-reported, which may have introduced reporting bias and influenced wound-healing outcomes.

## Conclusion

This study demonstrates that VSMP breast reduction is a safe and effective option for patients requiring large-volume reduction, including those with high BMI. Complication rates were low, with no hematomas or seromas, and aesthetic outcomes were highly satisfactory-over 80% of patients rated their results as “very good” or “excellent”. Despite the study’s small size, the findings support VSMP as a reliable first-line technique for macromastia, offering excellent aesthetic results, minimal complications, and high patient satisfaction, particularly in low-resource settings.

## Authors’ contributions

**Yahia Ahmed Alsiaghi and Mohammed Hasan Al. Shoaibi:** Writing – review & editing, Supervision, Methodology, Investigation, Conceptualization. **Mohaned Yahia Al-ajaly:** Visualization, Methodology, Formal analysis, Data curation, Resources. **Jehad Al-Mortada:** Writing – original draft, Visualization, Methodology, Investigation, Formal analysis, Data curation, Project administration, Conceptualization. **Aymen Mohammed Ghanem:** Writing –original draft, Investigation, Formal analysis, Data curation. **Ahmed A.S. AL-Magedi:** Writing – review & editing, Visualization, Software, Investigation.

## Declaration of generative AI and AI-assisted technologies in the writing process

Nothing to disclose.

## Availability of data and materials

Data used and/or analyzed during the current study are available from the corresponding author on reasonable request.

## Ethical approval

The study protocol was approved by the Ethics Committee of the Yemeni Council for Medical Specializations, Sana’a, Yemen (approval no IRB/YBMS/2022/047).

## Declaration of competing interest

The authors have no financial interest to declare in relation to the content of this article.

## Appendix Supplementary materials

Image, application 1

Image, application 2
