# Supplementary material for: Vertical scar reduction mammoplasty in massive macromastia: A prospective case series
Source: JPRAS Open. 2026 Jan 16;48:759–77. doi: 10.1016/j.jpra.2026.01.012 (PMC12918169; doi:10.1016/j.jpra.2026.01.012)
Supplement: Supplementary file 1 [file mmc1.docx]

**Questionnaire**

**Consent:**

Are you agreeing to the followings?

Your participation is voluntary and you can withdraw at any time.

All information collected will be kept confidential.

The results of this study may be published in scientific journals or presented at conferences. However, your personal information will not be identifiable.

**Demographic Data:**

Name……………………………… Age…………. Weight ……. Height …. BMI …. Marital status: single ………… married ………… divorced …….. widow ….

**Risk factors and comorbidities:**

1-Smoking: Yes ( ) No ( )

2-Chronic diseases: HTN ( ) DM ( ) others (………)

3-steroids use : Yes ( ) No ( )

4-Obesity: Yes ( ) No ( )

5-Overweight: Yes ( ) No ( )

**Presenting symptoms:**

1-Pain: neck ( ) back ( ) shoulder ( )

2-Intertriginous rash: Yes ( ) No ( )

3-Shoulder grooving: Yes ( ) No ( )

4-Asymmetry: Yes ( ) No ( )

5-Upper limb paresthesia: Yes ( ) No ( )

6-Sleep compromise: Yes ( ) No ( )

7-Social embracement: Yes ( ) No ( )

8-Others …………..

**Preoperative Characteristics :**

1-SN to Nipple Distance: Rt. Breast ( ) Lt. Breast ( )

2-Nipple to IMF Distance: Rt. Breast ( ) Lt. Breast ( )

3-Areolar diameter: Rt. ( ) Lt. ( )

4-Internipple Distance ………

**Intraoperative characteristics :**

1-Resected weight: Rt. Breast :……... Lt. Breast:……….

Postoperative characteristics :

1-Drain time of removal ( )

2-Length of stay in hospital in days ( )

**Surgical complications and aesthetic outcomes :**

**Early complications (within 30 days of operation) :**

1-Hematoma: Yes ( ) No ( )

2-Seroma: Yes ( ) No ( )

3-Wound dehiscence: Yes ( ) No ( )

4-Wound infection: Yes ( ) No ( )

5-Necrosis: Yes ( ) No ( )

If yes: necrosis of: wound edges ( ) NAC ( ) Fat necrosis ( )

**Late complications (by using standardized extended scheme of Ferreira):**

1-Volume (size): 0 (poor) 1 (fair) 2 (good)

2-Shape: 0 (poor) 1 (fair) 2 (good)

3-Symmetry: 0 (poor) 1 (fair) 2 (good)

4-Areola: 0 (poor) 1 (fair) 2 (good)

5-Scars: 0 (poor) 1 (fair) 2 (good)

**Revisions:**

No revision ( ) secondary revision ( ) tertiary revision ( )

**Patient satisfaction:**

poor ( ) good ( ) very good ( ) excellent ( )
